# New insights into the metastatic behavior after breast cancer surgery, according to well-established clinicopathological variables and molecular subtypes

**DOI:** 10.1371/journal.pone.0184680

**Published:** 2017-09-18

**Authors:** Oreste Claudio Buonomo, Emanuele Caredda, Ilaria Portarena, Gianluca Vanni, Augusto Orlandi, Claudia Bagni, Giuseppe Petrella, Leonardo Palombi, Paolo Orsaria

**Affiliations:** 1 Department of Surgery, Tor Vergata University Hospital, Rome, Italy; 2 Department of Biomedicine and Prevention, Tor Vergata University Hospital, Rome, Italy; 3 Department of Internal Medicine, Medical Oncology Unit, Tor Vergata University Hospital, Rome, Italy; 4 Anatomic Pathology, Department Biomedicine and Prevention, Tor Vergata University Hospital, Rome, Italy; University of North Carolina at Chapel Hill School of Medicine, UNITED STATES

## Abstract

Despite advances in treatment, up to 30% of patients with early breast cancer (BC) experience distant disease relapse. However, a comprehensive understanding of tumor spread and site-specific recurrence patterns remains lacking. This retrospective case-control study included 103 consecutive patients with metastatic BC admitted to our institution (2000–2013). Cases were matched according to age, tumor biology, and clinicopathological features to 221 patients with non-metastatic BC (control group). The median follow-up period among the 324 eligible patients was 7.3 years. While relatively low values for sensitivity (71%) and specificity (56%) were found for axillary lymph node (ALN) involvement as an indicator of risk and pattern of distant relapse, nodal status remained the most powerful predictor of metastases (OR: 3.294; CL: 1.9–5.5). Rates of dissemination and metastatic efficiency differed according to molecular subtype. HER2-positive subtypes showed a stronger association with systemic spread (OR: 2.127; CL: 1.2–3.8) than other subgroups. Classification as Luminal or Non-Luminal showed an increased risk of lung and distant nodal recurrence, and a decreased risk in bone metastases in the Non-Luminal group (OR: 2.9, 3.345, and 0.2, respectively). Tumors with HER2 overexpression had a significantly high risk for distant relapse (OR: 2.127) compared with HER2-negative tumors and also showed higher central nervous system (CNS) and lung metastatic potential (OR: 5.6 and 2.65, respectively) and low risk of bone disease progression (OR: 0.294). Furthermore, we found significant associations between biological profiles and sites of recurrence. A new process of clinical/diagnostic staging, including molecular subtypes, could better predict the likelihood of distant relapses and their anatomical location. Recognition and appreciation of clinically distinct molecular subtypes may assist in evaluation of the probability of distant relapses and their sites. Our analysis provides new insights into management of metastatic disease behavior, to lead to an optimal disease-tailored approach and appropriate follow-up.

## Introduction

Molecular subtype classification is a breakthrough in breast cancer (BC) research. Different pathological behaviors are classified according to a gene expression profile-validated immunohistochemical surrogate panel [1–2], which means that clinicians should consider distinct biological features before selecting appropriate therapeutic strategies. Mortality in women with estrogen receptor (ER)-positive tumors remains fairly constant over time, whereas the mortality in women with ER-negative tumors is initially higher than that in women with ER-positive disease, falling to a lower rate after 7–10 years [3–4]. However, our understanding of the different molecular phenotypes has been mainly limited to early BC, with an acceptance of different risks of relapse and responses to adjuvant therapies, deferring the question of whether the novel classification modifies the metastatic pattern for future exploration. Models of distant spread describe a complex interaction of seed and soil factors involving tumor circulation, proliferation, angiogenesis, and the microenvironment of the target tissue. Interestingly, among the major molecular subtypes, some preliminary associations have been identified. Lung and bone metastasis signatures have been reported, and human epidermal growth factor receptor 2 (HER2) and ER expression status has been associated with increased risk of spread to specific sites [5]. In general, triple negative breast cancer (TNBC) is associated with a worse prognosis than other stage-matched tumor phenotypes, partially reflecting tumor biology and partially a lack of validated target therapies for patients with this form of the disease. Metastatic TNBC is associated with considerably more visceral involvement compared with other BCs, with a dramatically increased risk of lung and central nervous system (CNS) as the first sites of recurrent disease [6]. Furthermore, baseline staging tests after a new diagnosis of BC based on subtype classification are the subject of debate. It is likely that conventional imaging would not necessarily detect all metastatic disease, with subclinical metastases missed simply because no or inappropriate imaging is performed. This diagnostic bias would be present for all specific patient subpopulations and would not affect the reported differences in the biological pathways and timing of distant relapse. Therefore, the aim of the present study was to determine the frequency and distribution pattern of metastases categorized by molecular subtypes in patients with newly diagnosed BC and to identify valuable clinicopathological variables associated with site of distant recurrence. The following analyses are presented to pool individual data from our series, in order to explore the relative survival of the major subtypes as classified using immunohistochemical markers, and to characterize their prognostic effects over time. Recognition and appreciation of these clinically distinct subgroups of BC may help us to predict distinct outcomes and may provide new insights into management of the disease. With increasing understanding of tumor biology, it is hoped that ongoing and future clinical trials will translate into improved outcomes for patients.

## Materials and methods

### Study design

We designed a case-control study, with cases and controls paired by age, age of onset, and duration of observation with a 1:2 case:control ratio. The risk of exposure in controls was assumed as 0.06 (6% of patients with BC develop one or more metastases). The alpha error and the power of the study were fixed at 5% and 80% respectively. Patients were enrolled according to defined eligibility criteria: same length of observation period between cases and controls; diagnosis, surgery, radiochemotherapy, and follow-up at the Tor Vergata University Hospital; and not lost to follow-up.

### Population

The study included 324 patients with newly diagnosed BC treated from January 2000 to July 2013 at Tor Vergata University Hospital. The median age of patients at enrollment was 65 years. The median age at disease onset was 58 years. Additional data are shown in Table 1.

**Table 1:** Description of case and control groups.

	Cases (103)	Controls (221)
***age at enrollment (****median)*	**65** (38–93)	**65** (40–93)
*(IQ)*	(IQ: 53–75)	(IQ: 55–76)
***age of onset of disease*** *(median)*	**57.8** (29–81)	**57.8** (30–88)
*(IQ)*	(IQ: 46.2–68)	(IQ: 46.9–67.3)
***time of observation in years*** *(median)*	**7.3** (1.8–14.7)	**7.2** (1.7–14.1)
*(IQ)*	(IQ: 4.4–10)	(IQ: 4.7–9.9)

IQ, interquartile range

Our dataset consisted of 103 metastatic BC cases (with distant disease progression) and 221 controls with no evidence of distant (metastatic) relapse after a median follow-up of 7.2 years (interquartile [IQ] range: 1.7 to 14.7).

All patients with invasive carcinoma received local excision and axillary sentinel node biopsy or axillary clearance. The following data were included in the analysis: pathological T stage, tumor grade (G) as lymph node status, and the expression of immunohistochemically defined biological markers (ER, progesterone receptor [PR], Ki-67, and HER2). The use of adjuvant/neoadjuvant systemic therapy was determined by the treating physician according to Italian guidelines. In particular patients received schedules containing anthracyclines (i.e. A-CMF[7], FEC 75[8]), anthracyclines and taxanes (AC-TXT[9] or TAC[10]) or without anthracyclines and taxans (i.e. CMF[11]).

### Methods

We identified intrinsic breast cancer subtypes according to the clinicopathological criteria recommended by the St. Gallen International Expert Consensus Report 2013 [12]. Patients were categorized based on the receptor status of their primary tumor as follows: luminal A (ER+ or PR+, and HER2-); luminal B HER2- (ER+, HER2- and at least one of Ki-67 “high” or PR “negative or low”); luminal B HER2+ (ER+, HER2-overexpressed or amplified, any Ki-67, any PR); HER2 (ER- or PR-, and HER2+), and basal (ER- or PR-, and HER2-). ER and PR status was determined using immunohistochemistry (IHC). Tumors were considered HER2-positive only if they were either scored 3+ by IHC (strong, complete membrane-staining in >30% of cancer cells) or showed HER2 amplification (ratio > 2) using fluorescence in situ hybridization (FISH). In the absence of positive FISH data, tumors scored 2+ using IHC were considered negative for HER2. Tumors were also classified as Luminal and Non-Luminal according to hormone receptor expression.

The Breast Cancer Outcomes Unit database was used to link clinical, pathologic, treatment, and outcome data, and the date of diagnosis and anatomic site were retrieved for up to the first five distant relapses. Distant relapse was defined as recurrences of BC occurring beyond the confines of the ipsilateral breast, chest wall, or regional lymph nodes. Sites of distant relapse were categorized as follows: brain (including choroid, CNS, pituitary gland, leptomeninges, and frontal sinus), liver, lung (including lymphangitic carcinomatosis), bone (including bone marrow), distant nodal (nodes beyond the ipsilateral axillary/supraclavicular internal mammary area), pleural/peritoneal (including skin outside of breast/chest wall, ovaries, spinal cord, eye, heart, and other organs not elsewhere classified), and unknown (patient known to have distant metastases but site or sites unknown).

Patient and tumor characteristics were compared across the five BC subtypes using the χ^2^ and Wilcoxon rank-sum tests for categorical and continuous variables, respectively. Cumulative incidence curves were estimated for each site of relapse according to competing risks methodology. These methods allow for the estimation of the cumulative incidence of an event of interest in the presence of competing events. Association between the site of relapse and breast cancer subtype was assessed in patients who experienced distant relapses using the χ^2^ test. This association was further examined in multivariate models using logistic regression with relapse to a specific site (yes or no) as the dependent variable and BC subtype and other patient/tumor characteristics included as covariates. All statistical analyses were carried out using SPSS (IBM SPSS V.23, Chicago, IL, USA). The study was approved by the institutional review board at the Tor Vergata University Hospital and conducted according to all current ethical guidelines. Written informed consent was obtained from all patients.

## Results

### T score

Concerning tumor size (pT), a greater proportion of T2 invasive BC (45.6%) occurred among cases compared with controls (27.9%), as well as a lower proportion of T1 (35.6% vs. 63.9%) (Table 2). T value distribution through univariable binary logistic analysis appeared strongly significant (p < 0.001), across the T progression (odds ratio [OR]: 1.896; confidence level [CL]: 1.397–2.573).

**Table 2:** Proportion of tumor size categories in case and control groups.

		*T score*					*Total*
		*0*	*1*	*2*	*3*	*4*	
***Cases***	n. (%)	3 (3.3%)	32 (**35.6%**)	41 (**45.6%**)	4 (4.4%)	10 (11.1%)	90 (100%)
***Controls***	n. (%)	4 (1.8%)	140 (**63.9%**)	61 (**27.9%**)	10 (4.6%)	4 (1.8%)	219 (100%)
***Total***	n. (%)	7 (2.3%)	172 (55.7%)	102 (33.0%)	14 (4.5%)	14 (4.5%)	309^a^ (100%)

^a^: Data not present for all patients

### G score

Among the tumors, 15.42% were scored as grade 1 (G1), 47.9% as grade 2 (G2), and 36.7% as grade 3 (G3). Moreover, although a higher proportion of G1 was observed in the group of patients free of distant recurrence, compared with those with metastatic disease (19.7% vs. 8.5%), no significant difference in the distribution of G2–G3 tumors was found between cases and controls (G2: 47.9% vs. 47.9%; G3: 43.7% vs. 32.5%) (Table 3). However, univariate binary logistic regression analysis revealed a growing risk (OR: 1.625; CL: 1.041–2.535 across the G progression) that was statistically significant (p: 0.03).

**Table 3:** Proportion of grading features in case and control groups.

		*G*			*Total*
		*1*	*2*	*3*	
***Cases***	n. (%)	6 (8.5%)	34 (47.9%)	31 (43.7%)	71 (100%)
***Controls***	n. (%)	23 (19.7%)	56 (47.9%)	38 (32.5%)	117 (100%)
***Total***	n. (%)	29 (15.4%)	90 (47.9%)	69 (36.7%)	188 ^a^ (100%)

^a^: Data not present for all patients

### Axillary lymph node

Axillary lymph node (ALN) involvement was significantly higher in metastatic cases than in control subjects (71.1% vs. 43.1%), and a lower incidence of N0 was observed (28.9% vs. 56.9%) (Table 4). Interestingly, this association was strong (OR: 3.25; 95% confidence interval [CI]: 1.9;5.5; p < 0.001) but there were a number of patients that did not show ALN involvement even in the presence of distant relapses (28.9%). Conversely, 43.1% of controls showed ALN involvement initially. Our analysis of these data follows in the Discussion section.

**Table 4:** Distribution of ALN involvement in case and control groups.

		*ALN*		*Total*
		*No*	*Yes*	
***Cases***	n. (%)	26 (**28.9%**)	64 (**71.1%**)	90 (100%)
***Controls***	n. (%)	120 (**56.9%**)	91 (**43.1%**)	211 (100%)
***Total***	n. (%)	146 (48.5%)	155 (51.5%)	301^a^ (100%)

^a^: Data not present for all patients. ALN, axillary lymph node

### Type of surgery

The extent of primary breast surgery according to standards and individualized concepts did not differ between the two study groups, resulting in a homogeneous rate of conservative and radical interventions. In case patients, there was a slightly higher incidence of radical surgery compared with that in controls (42.6% vs. 36.1%), but there was nonetheless a similar percentage of conservative surgery compared with that in patients free of distant disease recurrence (57.4% vs. 63.9%) (Table 5).

**Table 5:** Proportion of breast-conserving surgery (BCS) among molecular subtypes.

		*Molecular subtype*					*Total*
		*Lum A*	*Lum B-*	*Lum B+*	*HER2*	*Basal*	
***BCS***	***No***	34 (32.4%)	30 (39%)	16 (45.7%)	10 (52.6%)	9 (45%)	99 (38.7%)
	***Yes***	71 (67.6%)	47 (61%)	19 (54.3)	9 (47.4%)	11 (55%)	157 (61.3%)
***Total***		105 (100%)	77 (100%)	35 (100%)	19 (100%)	20 (100%)	256 ^a^ (100%)

^a^: Data not present for all patients. HER2, human epidermal growth factor receptor 2

### Type of chemotherapy

Patients were stratified on the basis of adjuvant chemotherapy. In total 174 patients (54% of 324 patients between cases and controls) underwent to adjuvant treatment after surgery: 83 (25,6%) received anthracyclines-based chemotherapy, 60 (18,5%) anthracyclines and taxanes-based chemotherapy while 31 (9,6%) patients received schedule without anthracycles (i.e. CMF). On 61 patients with HER2 positive-tumors 25 (41%) received adjuvant trastuzumab in addition to chemotherapy. All patients with positive hormone receptors received hormone therapy according to international guidelines.

The analysis of the impact of chemotherapy on the risk of develop distant metastases showed no significant differences between patients who did or did not underwent to adjuvant chemotherapy. In addition, there were not significant differences comparing different treatment schedules (anthracyclines *versus* anthracyclines and taxanes *versus* others schedules) and the risk to develop distant metastasis. In the HER2 positive tumors, adding trastuzumab to chemotherapy seems to defend patients for distant metastasis with a protective trend (OR: 0.9) but not statistically significant (p: 0.5).

### Molecular subtypes

The majority of patients had luminal A tumors (41.4%, 134 of 324), followed by luminal B (32.1%, 104 of 324), HER2-positive luminal B (12.3%, 40 of 324), basal (7.7%, 25 of 324), and non-luminal HER2-positive (6.5%, 21 of 324). The percentages categorized as Luminal and Non-Luminal were 85.8% (278 of 324) and 14.2% (46 of 324), respectively (Table 6).

**Table 6:** Distribution of molecular subtypes according to cases and controls.

		Molecular subtype					Total
		*Lum A*	*Lum B-*	*Lum B+*	*HER2*	*Basal*	
***Cases***	***n*. *(%)***	32 (31.1%)	35 (34%)	17 (16.5%)	11 (10.7%)	8 (7.8%)	103 (100%)
***Controls***	***n*. *(%)***	102 (46.2%)	69 (31.2%)	23 (10.4%)	10 (4.5%)	17 (7.7%)	221 (100%)
***Total***	***n*. *(%)***	134 (41.4%)	104 (32.1%)	40 (12.3%)	21 (6.5%)	25 (7.7%)	324 (100%)

HER2, human epidermal growth factor receptor 2

According to the impact of molecular subtypes on ALN involvement, the total metastatic rate of luminal B tumors was the highest (38.06%, 59 of 155), followed by those of luminal A (32.9%, 51 of 155), HER2-positive luminal B (11.61%, 18 of 155), non-luminal HER2-positive (9.03%, 14 of 155) and basal (8.38%, 13 of 155) subtypes (Table 7).

**Table 7:** Distribution of ALN involvement among molecular subtypes.

	Molecular subtype					Total
	*Lum A*	*Lum B-*	*Lum B+*	*HER2*	*Basal*	
**No ALN**	75 (59.5%)	38 (39.2%)	18 (50%)	4 (22.2%)	11 (45.8%)	146 (48.5%)
**Yes ALN**	51 (40.5%)	59 (60.8%)	18 (50%)	14 (77.8%)	13 (54.2%)	155 (51.5%)
**Total**	126 (100%)	97 (100%)	36 (100%)	18 (100%)	24 (100%)	301 (100%)

This distribution is statistically significant with a χ^2^ test (p: 0.006). ALN, axillary lymph node; HER2, human epidermal growth factor receptor 2

According to grouping of subtypes, more axillary metastases occurred in the Luminal group than in the Non-Luminal group (82.6%, 128 of 155 vs. 17.4%, 27 of 155) (Table 8).

**Table 8:** Distribution of ALN involvement according to luminal or non-luminal profiles.

	*Lum*	*Not Lum*	*Total*
**No ALN**	131 (89.7%)	15 (10.3%)	146 (100%)
**Yes ALN**	128 (82.6%)	27 (17.4%)	155 (100%)
**Total**	259 (86%)	42 (14%)	301 (100%)

The χ^2^ test for this distribution is not statistically significant (p: 0.074). ALN, axillary lymph node

In addition, specific analysis of locoregional lymph node status based on the risk of distant metastases and tumor biological profile revealed that 64% (9 of 14) of HER2-positive patients with axillary disease developed distant metastases compared with 25% (1 of 4) of patients with N0 disease. Furthermore, 42% of patients with tumors classified as luminal B (25 of 59) and 50% with tumors classified as HER2-positive luminal B tumors (9 of 18) with positive lymph nodes developed distant metastases, compared with 13% and 28% (5 of 38; 5 of 18) of patients with N0 disease, respectively. However, among patients with luminal A subtype, node positive disease constituted 33% (17 of 51) of cases, while node negative disease made up 16% (12 of 75) of cases.

In conclusion, more distant recurrences occurred in the Non-Luminal group with axillary disease, compared with the group with Luminal disease with the same locoregional spread (50%, 13 of 27 vs. 40%, 51 of 128).

### Metastases

Among the five intrinsic BC subtypes, non-luminal HER2-positive tumors had the highest total recurrence rate (52.3%, 11 of 21), followed by HER2-positive luminal B tumors (42.5%, 17 of 40), luminal B tumors (33.6%, 35 of 104), and basal tumors (32%, 8 of 25). Only 23.8% of patients with luminal A tumors (32 of 134) experienced distant cancer recurrence, a rate that differed significantly from the total recurrence rates of patients with HER2-positive luminal B (42.5%, 17 of 40) and non-luminal HER2-positive tumors (52.3%, 11 of 21) (Table 9).

**Table 9:** Number and percentage of distant metastases per molecular subtypes.

Molecular subtype	*n*.	*% for each subtype*
***Lum A***	32	23.9%
***Lum B-***	35	33.7%
***Lum B+***	17	42.5%
***HER2***	11	52.4%
***Basal***	8	32%
**Total**	103	

The rate of metastases was higher in the Non-Luminal group than in the Luminal group (41.3%, 19 of 46 vs. 30.21%, 84 of 278, respectively). However, given the greater frequency of Luminal subtype among the cases, the majority of patients who experienced distant relapse had positive expression of ER-related genes (81.5%, 84 of 103), information that is relevant in evaluation of the effects on outcome and cost-effectiveness of current operating strategies.

Evaluating the associated risk of distant metastases for each molecular subtype using a univariate binary logistic regression test, we observed that two of the five classes, luminal A and HER2, had statistically significant ORs of 0.5 and 2.5, respectively. These results demonstrate the lower tendency toward distant recurrences for luminal A and the higher tendency for HER2 tumor subtypes (Table 10).

**Table 10:** Associated risk of distant metastases among molecular subtypes.

*Molecular subtype*	OR (ExpB)	95% CI	p value*
***Luminal A***	**0.526**	**0.321–0.862**	**0.010**
***Luminal B HER2 neg***	1.134	0.690–1.864	0.620
***Luminal B HER2 pos***	1.702	0.866–3.346	0.120
***HER2***	**2.523**	**1.035–6.148**	**0.036**
***Basal***	1.011	0.421–2.424	0.981

*p value is calculated using χ^2^ test.

OR, odds ratio; CI, confidence interval; HER2, human epidermal growth factor receptor 2

Nevertheless, the classical binomial distribution of molecular subtypes, Luminal/Non-Luminal, does not lead to a final statistical significance versus the outcome of our study. Evaluation based on two categories according to HER2 overexpression revealed that both HER2-enriched and luminal/HER2 subtypes had a relatively high risk of distant metastases (OR: 2.127; 95% CL: 1.202–3.762; p: 0.009) (Table 11).

**Table 11:** Binomial distribution of molecular subtypes into luminal/non-luminal and HER2-positive/HER2-negative profiles in the case group compared to controls.

	*Lum*	*Not Lum*	Total	*HER2+*	*Not HER2+*	Total
***Cases***	84 (81.5%)	19 (18.5%)	103 (100%)	75 (72.8%)	28 (27.2%)	103 (100%)
***Controls***	194 (87.8%)	27 (12.2%)	221 (100%)	188 (85%)	33 (15%)	221 (100%)
***Total***	278 (85.8%)	46 (14.2%)	324 (100%)	263 (81.2%)	61 (18.8%)	324 (100%)

HER2, human epidermal growth factor receptor 2

According to the distant relapse rate among cases (n = 103), metastases were more frequent in bone (67%, 69 of 103) than in liver (40.8%, 42 of 103) and lung (36.9%, 38 of 103). The rates of distant nodal recurrence and CNS metastases were 22.3% (23 of 103) and 12.6% (13/103), respectively (Table 12).

**Table 12:** Frequency of metastases associated with sites of breast cancer relapse.

Metastases	Frequency (%*)
***Bone***	69 (67%)
***Liver***	42 (40.8%)
***Lung***	38 (36.9%)
***Distant Nodes***	23 (22.3%)
***Brain***	13 (12.6%)
***Total***	185

* The percentage is calculated based on the total number of cases (n = 103)

Specifically, after long-term follow-up, our case dataset included 39 patients with a single site (37.9%) of metastasis and 64 patients (62.1%) with multiple sites of metastases.

For patients with tumors defined as luminal A, luminal B, HER2-positive luminal B, non-luminal HER2-positive, and basal, the bone metastasis rates were 84.4%, 71.4%, 58.8%, 27.3%, and 50%, respectively (p: 0.007); the liver metastasis rates were 31.3%, 48.6%, 52.9%, 36.4%, and 25%, respectively (p: 0.401); the lung metastasis rates were 18.8%, 34.3%, 52.9%, 45.5%, and 75%, respectively (p: 0.19); the distant nodal metastasis rates were 9.4%, 20.0%, 29.4%, 45.5%, and 37.5%, respectively (p: 0.086); and the CNS metastasis rates were 9.4%, 5.7%, 23.5%, 36.4%, and 0%, respectively (p: 0.086) (Table 13).

**Table 13:** Frequency of distant organ involvement by each breast cancer subtype.

	Molecular subtype						χ^2^ test
	*LUM A*	*LUM B-*	*LUM B+*	*HER2*	*BASAL*	*Total*	*p value*
***BONE (%)***	27 (39.1%)	25 (36.2%)	10 (14.5%)	3 (4.3%)	4 (5.8%)	69 (100%)	0.007
*% per subtype*	*84*.*4%*	*71*.*4%*	*58*.*8%*	*27*.*3%*	*50%*	*67%*	
***LUNG (%)***	6 (15.8%)	12 (31.6%)	9 (23.7%)	5 (13.2%)	6 (15.8%)	38 (100%)	0.019
*% per subtype*	*18*.*8%*	*34*.*3%*	*52*.*9%*	*45*.*5%*	*75%*	*36*.*9%*	
***LIVER (%)***	10 (23.8%)	17 (40.5%)	9 (21.4%)	4 (9.5%)	2 (4.8%)	42 (100%)	0.401
*% per subtype*	*31*.*3%*	*48*.*6%*	*52*.*9%*	*36*.*4%*	*25%*	*40*.*8%*	
***BRAIN (%)***	3 (23.1%)	2 (15.4%)	4 (30.8%)	4 (30.8%)	0 (0%)	13 (100%)	0.034
*% per subtype*	*9*.*4%*	*5*.*7%*	*23*.*5%*	*36*.*4%*	*0%*	*12*.*6%*	
***Distant NODE n*. *(%)***	3 (13%)	7 (30.4%)	5 (21.7%)	5 (21.7%)	3 (13%)	23 (100%)	0.086
*% per subtype*	*9*.*4%*	*20%*	*29*.*4%*	*45*.*5%*	*37*.*5%*	*22*.*3%*	

HER2, human epidermal growth factor receptor 2

For tumors classified as Luminal and Non-Luminal, the bone metastasis rates were 73.8% and 36.8%, respectively; the liver metastasis rates were 42.8%, and 31.5%, respectively; the lung metastasis rates were 32.14%, and 57%, respectively; the distant nodal metastasis rates were 17.8%, and 42.1%, respectively; and the CNS metastasis rates was 10.7% and 21%, respectively.

These findings confirmed that Non-Luminal BC, including HER2-positive and basal-like, was a “protective” factor for bone metastases with a high significance, lowering the risk by 80% (OR: 0.2). Furthermore, the probability of lung and distal nodal metastases seemed to be more related to the Non-Luminal BC group, with a three times higher risk compared with the Luminal subtypes together (OR: 2.9 and OR: 3.345 respectively) (Table 14).

**Table 14:** Organ-specific metastatic risk according to binomial distribution of molecular subtypes into luminal/non-luminal profiles.

*Site of metastases*	OR	95% CI	χ^2^	p value
***Bone***	**0.207**	0.072–0.592	9.576	0.002
***Lung***	2.9	1.047–8.045	4.414	0.036
***Distant nodes***	3.345	1.150–9.736	5.253	0.022

OR, odds ratio; CI, confidence interval

With classification of tumors into two categories according to HER2 overexpression, both HER2-enriched and luminal/HER2 subtypes showed a relatively and significantly high risk of CNS and lung metastases (OR: 5.6 and OR: 2,65 respectively), while continuing to demonstrate their protective role in the development of bone metastases (Table 15).

**Table 15:** Organ-specific metastatic risk according to binomial distribution of molecular subtypes into HER2-positive/HER2-negative profiles.

*Site of metastases*	OR	95% CI	χ^2^	p value
***Bone***	**0.294**	0.119–0.728	7.352	0.007
***CNS***	**5.6**	1.649–19.023	8.870	0.003
***Distant nodes***	2.65	0.997–7.040	3.972	0.046

HER2, human epidermal growth factor receptor 2; OR, odds ratio; CI, confidence interval

## Discussion

Prediction of distant metastases is of paramount importance in the understanding and management of patients with breast cancer. The search for factors determining the natural history of the disease has interested numerous investigators in an attempt to obtain a deeper knowledge of this pathology and to define different groups of patients according to the probability of distant relapse [13–15].

### Association of clinicopathological factors and distant relapse rate

#### Tumor size

A number of clinicopathological factors affect the cancer-specific outcome: tumor size is an obvious marker of deep changes in cell biology and a strong predictor of BC survival [16–18]. Among node-negative patients, increasing tumor size has been associated with increased breast cancer-specific mortality [19]. In addition, the growth rate of the primary tumor has been correlated with a higher risk of ALN involvement, the most significant and durable prognostic factor for women with BC [20]. We identified a linear increasing trend in (OR: 1.9; CL: 1.397–2,573) distant relapse rates with univariable binary logistic analysis. However this well-established relationship does not appear to hold in a small subset of cancers with more aggressive phenotypes and pathways with higher risk of invasion, as previously shown by other researchers [21]. Although traditionally, distant spread has been considered a late event in tumor progression, recent studies suggest that for some tumors, acquisition of metastatic potential may occur early in cancer development, even in the absence of detectable primary tumors [22–23].

#### Histopathological grade

Genome-wide microarray-based expression profiling studies have unraveled several characteristics of BC biology and have provided further evidence that the biological features encapsulated by histopathological grade are important in determining tumor behavior [24]. Although there are compelling indications to suggest that grade assessment can accurately predict the intrinsic biological features for clinically relevant subgroups of invasive cancers, in the present study, after five years, the Nottingham Grading System showed a limited prognostic association with distant recurrence rate. However, these results provide evidence that it is possible to obtain significant additional information of prognostic value with the use of already implemented multifactorial indices such as ALN involvement or molecular subtyping of BC, and this may be of value in improving the prediction of outcomes for individual patients.

#### Nodal status

Even in the era of gene expression profiling, nodal status remains one of the primary prognostic discriminants in BC patients, and is of great value as an independent predictor of distant disease development, as we reported in our study. Although several institutional case series have differed in patient selection, follow-up, type of surgery, and adjuvant therapies, they have consistently shown that the percentage of positive lymph nodes is a significant indicator of survival in women with axillary metastases on final pathology [25]. In the present study, we showed a specific correlation pattern between lymphatic disease progression and increased risk of metastatic spread, hypothesizing that nodal status is still a potential signature of the intrinsic biological properties of a primary tumor. On the other hand, further research will be required to establish whether there are reproducible organ-distinct patterns of distant recurrence across the different molecular subtypes as well as the relationship with nodal involvement, since the probability of axillary metastasis appeared to be more strongly related to the luminal BC group, with a three times higher risk in patients with luminal B tumors, compared with patients with non-luminal HER2 tumors. A lower presentation of node-positive cases in the Non-Luminal class could be interpreted as metastatic involvement of other lymphatic stations, or as a different manner of cancer cell spread, but this should be confirmed with the results of overall and disease-free survival, regarding the relevance of biological profile classification on BC behavior in routine clinical practice.

However, the majority of the studies assessing HER2 in node-positive BC have consistently shown HER2 overexpression to be associated with a worse prognosis, a finding consistent with the relatively high frequency of distant relapse (64%) in the HER2-enriched subgroup with locoregional nodal spread described in this study, compared with HER2 node-negative tumors (25%) [26–27]. Although the hypothesis remains somewhat controversial, activation of this proto-oncogene has been proposed to correlate with the presence of lymph node metastases and, in cases when clinical follow-up was available, also a bad prognosis [28–31]. The present study reinforces the positive correlation with lymph node status. In fact, although it also provides a growth advantage in localized disease, this oncogene may need to be accompanied by additional modulating genetic events to exert its full aggressive behavior effect in progressive systemic disease. With regard to the molecular subtypes in our study, the lymph node involvement shows the trend reported in the literature. We found an OR > 3 for the association between ALN involvement and distant relapse. On the other hand, the prognostic values of nodal disease burden in determining BC metastatic behavior, show significant false positive and negative rates. Further explorations of the feasibility of these techniques, as well as correlation with pathology and patient outcomes, will be helpful in assessing the benefit of these procedures. Moreover, sentinel node biopsy, a more elementary test comparable to screening, seems to be of relative importance in clinical practice. However, assessment of nodal status, in association with the primary tumor characteristics, demonstrated a very strong OR in our study and a high significance according to the literature context.

As our study is limited by its small sample size and retrospective nature, the binomial distribution of molecular subtypes, Luminal vs. Non-Luminal, revealed a trend toward association with distant relapse among Non-Luminal profiles, although it did not reach statistical significance. On the other hand, the division into two categories according to HER2 molecular subtypes (HER2 and luminal B HER2+) showed an increased risk (OR: 2.127; 95% CI: 1.202–3.762) for disease progression, with emphasis on the virulence factor conferred by the HER2 molecule.

### Association of breast cancer subtypes and distant relapse rate

Undoubtedly, BC is a group of heterogeneous diseases with substantial variation in both molecular and clinical characteristics. Rapid progress has been made in understanding the diversity of BC, leading to a new molecular-driven integrated classification. The novel biological analysis integrates molecular and clinical landscapes of BC to define five clusters with distinct clinical outcomes and provide new insights into the management of the disease. Our findings have implications for the individualization of therapy, bringing us a step closer to the realization of personalized medicine in breast cancer, but also provide new evidence for exploring the underlying mechanism of molecular subtypes [32].

In our case-control study, 103 patients with metastatic BC grouped into different subtypes and according to the main clinicopathological variables, showed different rates of distant recurrence.

The results of the present investigation were in agreement with the findings of an earlier study, which reported that HER2-positive and basal tumors were associated with a higher tendency toward systemic disease than the remaining subtypes, but this failed to reach statistical significance (OR: 1.625; CL: 0.857–3.083; p: 0.135) [33]. However, amplification and/or overexpression of the HER2 oncogene, which belongs to the epidermal growth factor receptor (EGFR/HER) family, occurs in about 15% of invasive BCs, and is associated with increased cell proliferation and motility, increased angiogenesis, tumor invasiveness, and decreased apoptosis [34–35]. In particular, there is increasing recognition that HER2-positive BC is characterized by poor clinical prognostic features, which translate into aggressive tumor behavior, and importantly by experimental and clinical resistance to endocrine therapy [36]. Indeed, HER2 signaling is associated with younger age, high nuclear grade, greater lymph node involvement, and negative hormone receptor status, although approximately half of HER2-positive cases co-express ER [37]. Based on the above, clinically defined HER2-positive subtype is not biologically homogeneous, and further studies are warranted to prospectively confirm that the ER and HER2 pathways engage in crosstalk and thereby synergize in tumor progression or differential sensitivity to therapies [38].

### Association of breast cancer subtypes and sites of distant relapse

A further consideration is that the five major molecular subtypes of BC show different trends with regard to their ability to metastasize to different organs, and share biological features and pathways with their preferred distant metastatic sites [39].

#### Bone

In the current study, a greater than 70% cumulative rate of bone metastases was documented among luminal A, luminal B, or HER2-positive luminal B subtypes, making bone the most commonly diagnosed site of distant relapse. This high rate may point to the central role that the bone marrow plays as a common homing organ for metastatic BC cells, independent of the pattern of overt metastasis [40]. However, bone has been previously described as a preferred site of metastasis among ER-positive tumors, a finding confirmed in this report [41].

#### Liver

The liver is one of the most common metastatic sites of BC, with hepatic recurrences developing in 6%–25% of patients and being associated with poor prognosis [42]. The highest numbers of patients experiencing liver relapse were found in the HER2-positive luminal B group and the luminal B subtype, confirming that increased phosphorylation of HER2 with high Ki-67 positivity appear to be extremely important for the establishment of BC liver metastases and the prediction of visceral recurrence, but this failed to reach statistical significance (p: 0.401) [43]. On the other hand, ongoing trials are exploring whether the clinicopathological characteristics of the primary tumor of patients with hepatic relapse had an influence on outcome. For instance, Duan et al. showed that the BC subtype was an independent prognostic predictor for patients with BC metastases to the liver. Survival after liver metastases arising from basal BC was 21 months compared with 30, 32, and 41 months for patients with the HER2, luminal B, and luminal A subtypes; liver metastases from basal BC had the worst prognosis [44]. However, future clinical studies with large samples and a prospective design are expected to validate this hypothesis and increasingly frequent clinical problem.

#### Central nervous system (CNS)

BC constitutes the second most common cause of brain metastases, which are clinically associated with a significantly detrimental impact on survival [45]. Previous studies have reported a 25% to 34% rate of CNS relapse among patients with metastatic HER2-positive disease, which is consistent with the frequency of 36.4% in the HER2-enriched subgroup in this study but higher than the rate found for the HER2-positive luminal B group (23.5%) [46–47]. Furthermore higher rates of CNS metastasis among ER-negative/HER2-positive tumors compared with ER-positive/HER2-positive tumors were observed in a previous study, a finding confirmed in the present analysis [48]. These results likely confirm that specific factors related to the biology of HER2 overexpression and the evolving efficacy of BC treatment are likely to contribute to the growing incidence of brain metastasis in BC, in addition to the limitations on drug delivery imposed by the intact blood-brain barrier in early BC treatment [49].

#### Lung

For patients experiencing lung relapse, the tumors that expressed the lung metastasis signature were very often of the basal subtype, which agrees with similar findings reported by Minn et al., who described many genes involved in the disease cell microenvironment, and differentially expressed in both a mouse and primary tumor model [50]. Signature genes include the epidermal growth factor receptor ligand epiregulin, cyclooxygenase COX2, and metalloproteinases 1 and 2, which collectively facilitate the assembly of new tumor blood vessels, the release of tumor cells into the circulation, and the breaching of lung capillaries by circulating tumor cells to seed pulmonary metastasis [51]. These findings reveal how aggressive primary tumorigenic functions can be mechanistically coupled to greater lung metastatic potential, and how such biological activities may be therapeutically targeted with specific drug combinations.

### Association of breast cancer molecular relationships and sites of distant relapse

Taken together, in line with prior studies, our observations have demonstrated that the BC subtypes clearly show preferential sites of distant disease. Further explorations of the feasibility of these prognostic and predictive markers, as well as correlation with pathology and patient outcomes, will be helpful in assessing the benefits of this expanding knowledge. However, the current report demonstrates important differences in metastatic behavior among the BC subtypes as defined by a panel of immunohistochemical markers, which may lead to an optimal disease-tailored approach.

The independent class analysis shows a tendency of some biomolecular variants toward exclusive pathological locations, with a clear trend toward preferential homing sites, although this finding was not statistically significant. Moreover, further investigations according to hormone receptor profile (Luminal or Non-Luminal tumors), and in relation to HER2 overexpression (HER2-positive luminal B/non-luminal HER2-positive vs. luminal A, luminal B, and basal), produced the most interesting results. These findings confirmed that Non-Luminal BC, including HER2 and basal-like, was a “protective” factor for bone metastases with a high significance, lowering the risk by 80% (OR: 0.2), and highlighting that the Luminal subtype (especially Luminal A) is responsible for almost all bone distant recurrences. Furthermore, the Non-Luminal tumors metastasize more frequently to lung (OR: 2.9) and distal nodal sites (OR: 3.34).

In addition, the classification of BC into HER2-positive or -negative subtypes shows among HER2-overexpressed tumors the same trend for bone metastases (thus exploring the implication that luminal A tumors were significantly associated with bone metastases, OR: 0.294), as well for distant nodal relapse rate (OR: 2.65) although with a less significant result, but with a relevant higher incidence of CNS metastases, OR: 5.6 (p: 0.003).

Thus, further investigation into the molecular mechanisms of these relationships may provide substantial clinical utility, since a better understanding of the risk of locoregional and distant tumor recurrence would be of benefit in therapeutic decision-making and in conducting appropriate follow-ups.

Our current study has several inherent limitations. Classification according to ER, PR, HER2, and Ki-67 status and grade are only approximations of genotype based molecular BC subtypes, and our conclusions do not necessarily apply to genotype-based subtypes. Detailed patient and tumor information that may have influenced treatment decisions were not available from the cancer registry database. Important factors regarding family history, genetic testing results, tamoxifen use, systemic chemotherapy, and mammographic findings were not available from this database. Another possible study limitation is the inability to report on the detailed pathology, staging, and outcomes for women referred to our institution; however, additional explorations of the feasibility of these profiling practices will be helpful in assessing the benefit of these classifications. Despite these potential limitations, we were able to acquire data from a large number of cases, and the power of the data appears to be high. Further studies will be required to confirm the findings based on these new definitions.

## Conclusion

Our study explored the validity of ALN involvement for distant relapses, finding relatively low values for sensitivity (71%) and specificity (56%). However, ALN involvement remained the most powerful predictor of metastases (OR: 3.294; CL: 1.9–5.5). The HER2 molecular subtype and the classes overexpressing HER2 (luminal B HER2+ and HER2 type) showed a stronger association with distant metastases (OR: 2.523, CL: 1.035–6.148 and OR: 2.127, CL: 1.2–3.8, respectively) in comparison with other subtypes. Finally, we found significant associations between molecular subtypes and the site of recurrence. Thus, a new process of clinical/diagnostic staging, including molecular subtypes, could better predict the likelihood of metastases and their anatomical site, e.g., classes overexpressing HER2 were strongly associated with brain metastases (OR: 5.6; CL: 1.6–19.0).

In conclusion, the observations reported here indicate that the five major molecular subtypes of breast cancer are not only distinct with regard to primary tumor characteristics, tumor aggressiveness, and response to certain types of chemotherapy, they are also clearly different with regard to their ability to metastasize to distant organs. Numerous prognostic factors in BC risk for relapse have been established, including tumor size, nodal status, hormone receptor status, and grade, but recognition and appreciation of these clinically distinct molecular subgroups may be helpful in predicting tumor behavior and guiding practice, with the awareness that the management of each patient must be considered in the context of that individual’s unique presentation. As more is understood regarding tumor biology, it is hoped that ongoing and future clinical trials will translate into improved outcomes for patients. Furthermore, the recognition of subtype-specific differences in short- and long-term prognosis will inevitably lead to tailored follow-up programs after completion of primary therapy, in order to characterize their prognostic effect over time as well as to prevent over- and under-treatment in the management of the disease.

## Supporting information

S1 Table Minimum dataset. (XLS)
